# CRISPR-based bioengineering in microalgae for production of industrially important biomolecules

**DOI:** 10.3389/fbioe.2023.1267826

**Published:** 2023-10-26

**Authors:** Dhananjay Dhokane, Arshi Shaikh, Anu Yadav, Nandinee Giri, Anindya Bandyopadhyay, Santanu Dasgupta, Bhaskar Bhadra

**Affiliations:** Synthetic Biology Group, Reliance Industries Ltd., Navi Mumbai, India

**Keywords:** bioactives, biomolecules, bioengineering, CRISPR/Cas9, microalgae, synthetic biology

## Abstract

Microalgae, as photosynthetic organisms, have the potential to produce biomolecules for use in food, feed, cosmetics, nutraceuticals, fuel, and other applications. Faster growth rates and higher protein and lipid content make microalgae a popular chassis for many industrial applications. However, challenges such as low productivity and high production costs have limited their commercialization. To overcome these challenges, bioengineering approaches such as genetic engineering, metabolic engineering, and synthetic biology have been employed to improve the productivity and quality of microalgae-based products. Genetic engineering employing genome editing tools like CRISPR/Cas allows precise and targeted genetic modifications. CRISPR/Cas systems are presently used to modify the genetic makeup of microalgae for enhanced production of specific biomolecules. However, these tools are yet to be explored explicitly in microalgae owing to some limitations. Despite the progress made in CRISPR-based bioengineering approaches, there is still a need for further research to optimize the production of microalgae-based products. This includes improving the efficiency of genome editing tools, understanding the regulatory mechanisms of microalgal metabolism, and optimizing growth conditions and cultivation strategies. Additionally, addressing the ethical, social, and environmental concerns associated with genetic modification of microalgae is crucial for the responsible development and commercialization of microalgae-based products. This review summarizes the advancements of CRISPR-based bioengineering for production of industrially important biomolecules and provides key considerations to use CRISPR/Cas systems in microalgae. The review will help researchers to understand the progress and to initiate genome editing experiments in microalgae.

## Introduction

**FIGURE 1:**
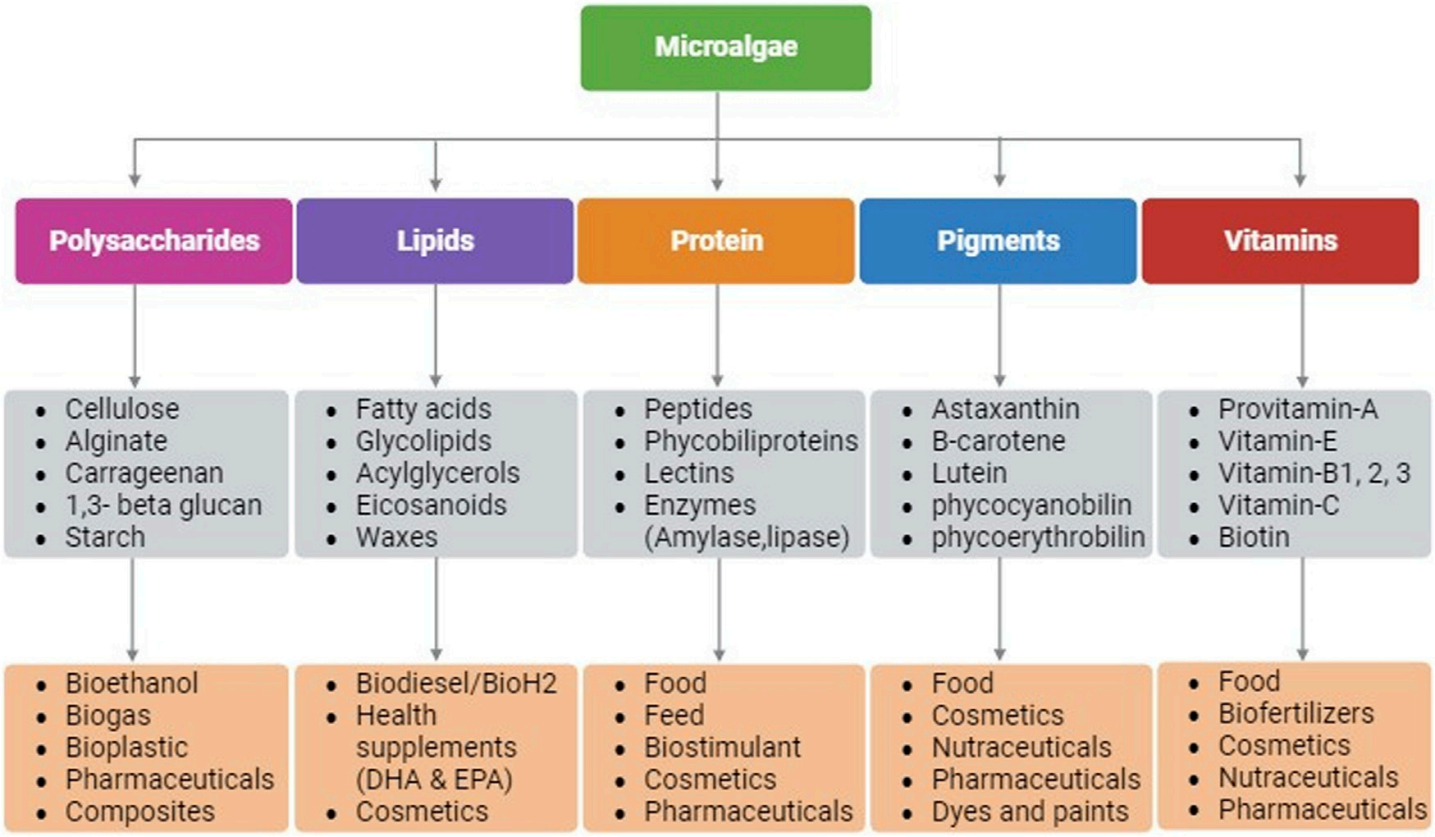
Schematic representation of biomolecules that are produced in microalgae and their applications. Created with BioRender.com.

Microalgae as photosynthetic cell factories are gaining attraction for several industrial applications due to their rapid growth rate, higher photosynthetic efficiency, ability to produce higher biomass, and complex metabolites (Kumar et al., 2020). Owing to the taxonomic and inherent biochemical diversity among microalgal species, production of bioactive molecules for applications in pharmaceuticals, nutraceuticals, personal care, dietary supplements, biofuels, medicine, food, feed, etc. are being continuously investigated (Kumar et al., 2020). Microalgae synthesize compounds such as proteins, lipids, polysaccharides, pigments, and vitamins that have diverse applications (Figure 1) (Dolganyuk et al., 2020). These compounds are known to possess anticancer, anti-inflammatory, antimicrobial, and antioxidant properties making microalgae a suitable host for pharmaceutical and nutraceutical applications. In addition, microalgae are exploited for biotechnological applications such as, for production of recombinant proteins/peptides, monoclonal antibodies, and vaccines (Khavari et al., 2021). Moreover, their lipid accumulating ability (20%–70% cell dry weight) make microalgae a promising candidate for biofuels and/or nutraceuticals depending upon their fatty acid composition (Kumar et al., 2020).

The increase in emissions of greenhouse gases (GHGs) in the environment due to extensive use of fossil fuels for energy has resulted in climate change, worldwide. Carbon dioxide (CO_2_) contributes to approximately 60% of total GHGs emissions globally (Solaymani, 2019). The levels of CO_2_ in the environment are continuously increasing and are impacting every form of life on the planet earth. A report from National Oceanic and Atmospheric Administration (NOAA) global monitoring lab shows that in the year 2022, the global average CO_2_ level was 417.06 ppm, hitting a new record. Increasing CO_2_ levels in the atmosphere is raising the temperature every year. NOAA also observed that in 2021, CO_2_ alone was responsible for about two-third of the total heating influence of all human-produced greenhouse gases (https://gml.noaa.gov/ccgg/trends/). To mitigate the adverse effects of CO_2_ emissions, lot of efforts are put forth globally to either reduce emissions or to sequester CO_2_. The United Nations has mandated achieving carbon neutrality by 2050. Carbon neutrality is balancing carbon emissions with carbon absorption to achieve a net zero carbon emissions in atmosphere. Carbon sequestration is the key process in achieving carbon neutrality. Globally, numerous efforts are concentrated on developing various carbon capture technologies; however, with little success due to complex economics (Sadvakasova et al., 2023). Therefore, biological sequestration of carbon would more likely be the method of choice to fix atmospheric CO_2_ sustainably.

Microalgae are phototrophic organisms that possess carbon accumulation and metabolic capabilities for CO_2_ sequestration (Sadvakasova et al., 2023). Microalgae can fix CO_2_ and convert solar energy into biomass at higher efficiencies (8%–10%) than land plants (Benedetti et al., 2018). They can serve as means of natural carbon sink and help in the reduction of CO_2_ burden globally. It has been reported that microalgae photosynthetically capture approximately 100 gigatons of CO_2_ per annum (Peters et al., 2017). This clearly demonstrates that microalgae are invaluable resources for sequestering atmospheric CO_2_ and generating biomass at industrial scale. To produce 1 kg of microalgal biomass, approximately 1.83 kg of CO_2_ is captured and fixed (Sarwer et al., 2022). The microalgal biomass acts as an excellent sustainable feedstock for renewable energy and valuable products for diverse industrial applications. The microalgal biomass consist of high value biomolecules that are in high market demand for applications in nutraceuticals, cosmetics, pharmaceuticals, food, and feed.

Despite the numerous industrial applications of microalgae, there are many challenges associated with exploiting microalgae to their fullest potential. The major challenge is to improve the microalgal productivity and product titers to make the production processes commercially viable. However, altering only the growth conditions does not guarantee improved productivities and titers of targeted products (Sreenikethanam et al., 2022) Genetic engineering has potential to alter the genetic constitution of a strain, thereby generating strains with higher growth rates, better photosynthetic efficiencies, and higher product titers. Genetic engineering using genome editing tools like CRISPR/Cas facilitates precise and targeted genetic manipulations. CRISPR/Cas systems are employed in microalgae for targeted gene knockouts and knockins, multiplex gene targeting, and modulation of gene expression, to produce industrially relevant strains (Jeong et al., 2023). CRISPR interventions in microalgae has generated strains with improved lipid and pigment contents, triacylglycerol productivity, lipid accumulation, efficient CO_2_ sequestration capabilities (Asadian et al., 2022; Lee et al., 2022; Song et al., 2022). Likely, CRISPR/Cas systems will facilitate rapid development of strains with higher lipid productivities for biodiesel production, higher pigment contents for applications in food, cosmetics and nutraceuticals, biomolecules of interest and high performing strains with industrially important traits. This review provides a holistic understanding of the progress that has been made in algal genome editing, how CRISPR/Ca9 systems has been utilized, for generating high performing algal strains for various industrial applications.

## Microalgae as a rich source of bioactives

Microalgae are excellent sources of diverse pigments, including carotenoids, xanthophylls, and phycobiliproteins (Saide et al., 2021). Phycobiliproteins are accessory light-harvesting pigment complexes of the algal photosynthetic machinery. They are made up of apoproteins (α, β, and γ subunits) and chromophores (phycocyanobilin, phycoerythrobilin, phycourobilin, and phycoviolobilin) linked by thioether bond and are found in red microalgae (e.g., *Porphyridium* spp. and *A. platensis*) (Ji et al., 2023). These are brightly colored water-soluble proteins and have numerous applications in food, cosmetic and biomedical industry. These proteins are known to act as antioxidants, boost the immune system, possibly lower the risk of heart diseases, prevent cancers, and protects against age-related diseases such as multiple sclerosis, and cataract (Hamouda and El-Naggar, 2021).

Fucoxanthin, a xanthophyll has shown potential in food, feed, health, and cosmetics applications. It exerts an antiobesity activity by modulating the increase of reactive oxygen species (ROS) and by down regulating lipid metabolism genes (Saide et al., 2021). Fucoxanthin has shown to reduce plasmatic and hepatic triglyceride concentrations and positively influences cholesterol-regulating enzymes, such as 3-hydroxy-3-methylglutaryl coenzyme A reductase and acyl-coenzyme A (D’Orazio et al., 2012). Microalgal-derived β-carotene is used as “natural” food additive with *Dunaliella salina* containing the highest amount and is used commercially in food, feed, supplements, and cosmetics applications. β-carotene exhibits various therapeutic potential, such as reduced risk of disease via modulating cell signaling pathways, antioxidant activity, nutritional value as provitamin A, peroxyl radicals scavenging activity, skin protective effects, and restoring hepatic enzymes (e.g., catalase, peroxidase, and superoxide dismutase) activity to protect vital organs against xenobiotic and other damages (Saide et al., 2021; Ampofo and Abbey, 2022). Astaxanthin exhibits great antioxidant activity and is commercially produced from microalgae *Haematococcus* spp. for use as colorant in feed industry, dietary supplement, in the prevention of some human pathologies, such as skin UV-mediated photo-oxidation, inflammatory processes, diabetes, and even cancer. Microalgal lutein protects cells from ROS damage under stress conditions and has potential role in preventing or ameliorating age-related macular degeneration, prevention of certain cancers, and for the protection of skin from UV-induced damage. Lutein has been extensively used as a feed additive and food coloration agent in industry (Saide et al., 2021; Ampofo and Abbey, 2022).

Microalgae are naturally abundant in phenolic compounds that are involved in various physiological processes. These compounds range from simple aromatic rings to more complex molecules and comprising flavonoids, phenolic acids, tannins, lignans or coumarins (Del Mondo et al., 2022). Polyphenols from microalgae exhibit extensive beneficial biological properties, including antioxidant, anticancer, antimicrobial, anti-inflammatory, antidiabetic, antiviral, and cardioprotective activities. *Tetraselmis suecica*, *Isochrysis* spp., *Chlorella vulgaris*, and *Phaeodactylum tricornutum* are the industrially relevant microalgae having highest antioxidant capacities and can be explored as potential new sources of natural antioxidants (Li et al., 2007; Hajimahmoodi et al., 2010; Saide et al., 2021).

Algal polysaccharides are mainly explored because of their bioactive properties for pharmaceutical, nutraceutical and biomedical applications (Patel et al., 2022). These polysaccharides are used as stabilizers and emulsifiers in food, feed, and pharmaceuticals. The microalgal polysaccharides are known to possess antiviral, antibacterial, antioxidant, anti-inflammatory, and immunomodulatory activities (de Jesus Raposo et al., 2014). In pharmaceutical and biomedical industry, polysaccharides like alginates, fucoidans, ulvans, carrageenans, and chitin are continuously explored. Polysaccharides from microalgae offer numerous advantages over synthetic polymers such as, safety, stability, hydrophilicity, biocompatibility, biodegradability, chemical modifiability, and biocompatibility. These properties enable them to be used for wide range of applications like, preparation of pharmaceutical materials, drug release agents, and plasma substitutes (Patel et al., 2022).

Microalgae are the factories for valuable lipids with approximately 25% of their dry weight, for example, fatty acids, polar lipids, oxylipins, and steroids, with promising applications in nutraceutical, pharmaceutical, cosmeceutical, and biofuel sectors. Microalgal lipids can be used in the prevention and treatment of several human pathologies, including anticancer, antioxidant, and anti-inflammatory activities, as well as treatment of diabetes. *Chlorella* spp. is found to have the most lipid content and is exploited commercially (Saide et al., 2021). Microalgae forms the best resource for production of third and fourth generation of biofuels with the ability to produce biodiesel, bioethanol, biohydrogen, etc. (Saide et al., 2021; Ampofo and Abbey, 2022). Higher lipid accumulation in their biomass along with great environmental adaptability for growth has a major advantage over other feedstock for microalgal biofuel production (Ampofo and Abbey, 2022). The major classes of microalgae suitable for biofuel production include Bacillariophyceae, Chlorophyceae, Eustigmatophyte, Chrysophyceae, Haptophyceae (Prymnesiophyceae), and Cyanophyceas, of which Chlorophyceae group is the most favorable for biodiesel production (Saide et al., 2021). Generally, most microalgal species have approximately 30% lipid content of their dry biomass with some species having even more lipid content, such as *Nannochloris* spp. (56%), *Chlorella* spp. (53%), and *Neochloris oleoabundans* (65%). However, growth rate decreases with higher oil-producing strains (Saide et al., 2021; Ampofo and Abbey, 2022). This lipid extracted from microalgae is then used for biofuel production. Additionally, neutral lipids or triacylglycerols are the major stored forms of lipids which can be esterified with C16 and C18 profiles and are proven to be ideal for biofuel production (Ampofo and Abbey, 2022).

Microalgae can produce phytosterols that have been used as additives in food products and have gained attention due to their reduced cholesterol concentration in blood, thereby preventing cardiovascular disorders (Saide et al., 2021; Ampofo and Abbey, 2022). Some species, such as *Isochrysis galbana*, *Nannochloropsis* spp., and *Phaeodactylum tricornutum*, have phytosterol content ranging from 7 to 34 g/kg (Ryckebosch et al., 2014); *Pavlova lutherie*, *Tetraselmis* spp. M8 and *Nannochloropsis* spp. BR2 may have phytosterol ranging 0.4%–2.6% dry weight, while 5.1% dry weight of phytosterol could be achieved for *P. lutherie* (Ahmed et al., 2015).

Microalgae are rich source of sustainable proteins, consisting of upto 70% of total protein content based on dry weight basis. *Arthrospira* spp., *Chlorella* spp., *Aphanizomenon* spp., and *Nostoc* spp., are known to have very high protein content. Microalgal proteins contain all the essential amino acids and possess balanced total amino acid profiles (Lucakova et al., 2022). Microalgal proteins are known to have unique physicochemical and technofunctional properties that can withstand diverse and harsh environmental conditions. Therefore, microalgal proteins are continually explored for new food and feed formulations (Acquah et al., 2021). Microalgal proteins and peptides have different bioactivities such as, antioxidant, anticancer, antihypertensive, antiatherosclerotic, anti-UV radiation, and antiosteoporosis (Saide et al., 2021; Ampofo and Abbey, 2022). Dermochlorella, an oligopeptide purified from the microalgae *Chlorella vulgaris*, has been widely used for skin treatments in the biomedical field (Martins et al., 2014).

## CRISPR/Cas as a genome engineering tool

CRISPR/Cas is presently emerging as one of the most promising genome editing tools for various organisms. The CRISPR locus was first discovered in the genome of *E. coli* in 1987 (Ishino et al., 2018) as a form of adaptive immunity against invading foreign DNA sequences of various viruses. It consists of an array of direct repeated sequences interspersed with short spacer sequences. The short spacer sequences are transcribed and processed into crRNAs (or guide RNAs) that in turn bind to effector nucleases (Cas proteins). This Cas ribonucleoprotein complex is then directed to the target DNA sequence (i.e., the sequence complementary to guide RNA) and owing to the nuclease activity of Cas protein, the target DNA is cleaved into shorter DNA fragments (Jeon et al., 2017; Ishino et al., 2018).

The ability of Cas nuclease to initiate double stranded breaks (DSBs) in the genomic DNA led to its usage as a genome editing tool in eukaryotic cells in 2013 (Cong et al., 2013; Mali et al., 2013). The DSBs created by Cas proteins are subsequently repaired using nonhomology mediated end joining machinery of the host cells, thereby creating indel (insertion/deletion) mutations at the target site. These indel mutations often result in creating knockout mutants of the target gene.

Strikingly, Cas nucleases are not the first endonucleases to be used as a genome editing tool. Zinc finger endonucleases as well as TALEN endonuclease have also been used to create targeted knockout mutants (Bibikova et al., 2002; Gaj et al., 2013; Zhang et al., 2013). However, the considerable surge in the popularity of the CRISPR/Cas system as a genome editing tool can be attributed to the fact that guide RNAs provide high degree of accuracy and flexibility to target any part of host genomic DNA sequence as per the requirement. In addition, with each passing day, different Cas proteins with varying degree of endonuclease activity are being discovered in various organisms (Kim et al., 2017a; Kim et al., 2017b). As a result, Cas endonucleases having better accuracy (with less off-target endonuclease activity) can be used for enhanced “targeted genome editing.” Till date, Cas9 isolated from *Streptococcus pyogenes* is the most used nuclease for CRISPR/Cas mediated genome editing (Ishino et al., 2018). CRISPR/Cas tool is used to create targeted knockout mutants, integrations of gene(s) at desired location within the genomes, replacement of nonfunctional alleles, transcriptional regulations, and many more applications. Although CRISPR-based tools open a plethora of opportunities to selectively edit the genomes of various species, the technique in microalgae as compared to other organisms is still in its infancy.

CRISPR-based multiplexed gene knockout and knockin has been explored to manipulate target genes and CRISPR interference or activation (CRISPRi/a) has enabled the modulation of complex metabolic pathways and regulatory networks. Here we have listed the CRISPR-based bioengineering efforts applied in several algal species for the production of biomolecules of industrial importance (Table 1).

**TABLE 1:** Examples of CRISPR-based bioengineering in different microalgal species to produce industrially important biomolecules.

Strain	Target gene modified	Type of modification	Type of nuclease	Outcomes	References
*Chlamydomonas reinhardtii*					
CC-4349	*CpFTSY,* zeaxanthin epoxidase (*ZEP*)	Knockout	Cas9	Mutant strain capable of constitutively producing 13-fold zeaxanthin with improved photosynthetic productivity, greater biomass accumulation observed under high light growth conditions	Baek et al. (2016)
CC-400	phosphoenolpyruvate carboxylase isoform 1 (*CrPEPC1*)	CRISPRi	dCas9	*CrPEPC1* downregulated strains showed increase in lipid content by 28.5% dry cell weight basis compared to WT	Kao and Ng (2017)
CC-4349	zeaxanthin epoxidase (*ZEP*)	Knockout	Cas9	Mutant strain exhibited significantly higher zeaxanthin content (56-fold) and productivity (47-fold) than the WT without the reduction in lutein level	Baek et al. (2018)
CC-4349	phospholipase A2 (*PLA2*)	Knockout	Cas9	Lipid productivities of phospholipase A2 knockout mutants increased by approximately 64.25%	Shin et al. (2019)
CC-4349	*ELT*	Knockout	Cas9	Higher lipid accumulation in mutant strain compared to WT and noticeable shift in fatty acid composition with an increase of approximately 27.2% in the C18:1 proportion making it suitable for biofuel production	Nguyen et al. (2020)
CC-4349	Zeaxanthin epoxidase (*ZEP*), lycopene epsilon cyclase (*LCYE*)	Knockout	Cas9	Zeaxanthin yield of double knockout mutant (*ZEP* and *LCYE*) strains were 60% higher. Zeaxanthin yield reported was (5.24 mg/L) compared to single ZEP mutant	Song et al. (2020)
CC-4349	ADP-glucose pyrophosphorylase (*AGP*), zeaxanthin epoxidase (*ZEP*)	Knockout	Cas9	Double knockout strains (*AGP* and *ZEP*) demonstrated 81% higher oil productivity along with same zeaxanthin and lutein concentrations as that of WT	Song et al. (2022)
UVM11	Bacterial *phytase* gene	knockin	Cas9	Showed site specific integration of bacterial *phytase* gene which clearly demonstarted that complex recombinant proteins and novel synthetic biomolecules can be successfully produced in food grade organism *C. reinhardtii*	Zadabbas Shahabadi et al. (2023)
*Phaeodactylum tricornutum*					
CCMP2561	*CryP*	Knockout	Cas9	Knockout showed increase in fucoxanthin content by 1.29-fold compared to WT	Yang et al. (2022)
Pt1 wild type (WT)	*ptACSL3*	Knockout	Cas9	*PtACSL3* mutants showed altered FA profiles in two galactoglycerolipids and phosphatidylcholine (PC) with significantly reduced distribution of 16:0 and 16:1	Hao et al. (2022)
CCAP 1055/1, & CCMP2712	Violaxanthin de-epoxidase (*VDL*2) and zeaxanthin epoxidase (*ZEP*1)	Knockout	Cas9	Demonstrated the role of two genes in Fucoxanthin biosynthesis. Knockout mutants of *VDL*2 and *ZEP*1 did not produce fucoxanthin	Bai et al. (2022)
*P. tricornutum* Bohlin	enoyl CoA hydratase (*PtECH*)	CRISPRi	dCas9	The *ECH* knockdown mutants exhibited an enhanced lipid accumulation relative to WT. The mutants showed higher photosynthetic efficiency, but impaired growth compared to WT	Guo et al. (2023)
*Nannochloropsis* spp.					
*N. gaditana* CCMP1894	*ZnCys*	Attenuation and Knockout	Cas9	Attenuated mutants of *ZnCys* demonstrated double the strain’s lipid productivity (∼5.0 g/m^2^/d) compared to WT (∼2.5 g/m^2^/d) and retained the ability to grow and fix CO_2_ at levels nearly equivalent to those of the WT strain	Ajjawi et al. (2017)
*N. gaditana* CCMP1894	Acyl-CoA oxidase (*Aco1*)	Knockout and Knockin	Cas9	Knockout mutant strains showed double the lipid productivity compared to WT	Verruto et al. (2018)
*N. salina* CCMP1776	Three cellulose synthase genes (cesA1, cesA2, cesA4)	Knockout	Cas9	Cell wall thickness and cellulose content were reduced in *cesA1* mutant but not in *cesA*2 or *cesA*4 cells. *CesA*1 mutation resulted in a reduction of chrysolaminarin and neutral lipid contents, by 66.3% and 37.1%, respectively, but increased the soluble protein content by 1.8-fold. Thinned cell wall cells were susceptible to mechanical stress, resulting in a 1.7-fold enhancement of lipid extractability	Jeong et al. (2020)
*N. gaditana* CCMP526	beta-glucan synthase (*BGS*), transglycosylase (*TGS*)	Knockout	Cas9	The generated knockout mutants showed ∼5-fold lower accumulation of soluble carbohydrate (β-1,3-glucose oligomers) following nitrogen starvation compared to WT, without any observed growth defect. The *TGS* knockout mutants showed 25%–40% (dry cell weight) increases in total fatty acids	Vogler et al. (2021)
*N. salina* CCMP1776	Δ12-fatty acid desaturase (*FAD12*)	Knockin	Cas9	The targeted knockin mutants of *FAD12* showed four-fold higher production of linoleic acid and 1.5-fold increase in eicosapentaenoic acid	Ryu et al. (2021)
*N. oceanica* IMET1	bZIP-family regulator *NobZIP77*	Knockout	Cas9	The *NobZIP77* knockout mutants under nitrogen deprivation showed similar growth like WT, with 3 times more triacylglycerol productivities	Zhang et al. (2022)
*Chlorella* spp.					
*C. vulgaris* FSP-E	Omega-3 fatty acid desaturase (*fad*3)	Knockout	Cas9	Knockout mutants of *fad*3 showed 46% (w/w) higher lipid content over WT	Lin and Ng (2020)
*C. sorokiniana* UTEX 1602	—	CRISPRa/i [Adaptive Single Guide Assisted Regulation DNA (ASGARD)]	dCas9	Gene regulation via CRISPRa-VP64 (CRISPRa) enhanced the protein contents up to 60% (w/w) of dry cell weight, where the highest concentration was 570 mg/L, while CRISPRi-KRAB (CRISPRi) with ASGARD increased protein content to 65% and lipid formed in the range of 150–250 mg/L (WT: 150 mg/L)	Lin et al. (2022)
*Parachlorella kessleri* NIES-2152	Three genes, calcium-dependent membrane targeting (9934_t), duplicated mannanases 1 (8741_t), plastidic ATP/ADP translocase (9067_t)	Knockout	Cas9	The knockout mutants of plastidic ATP/ADP translocase showed >30% more lipid productivity compared to WT strain under dark light cycle	Kasai et al. (2023)
*Tetraselmis spp.*					
*Tetraselmis* spp. KCTC12432BP	ADP-glucose pyrophosphorylase (AGP)	Knockout	Cas9	*AGP* mutants showed enhanced lipid production. Fatty acids in the *AGP* mutants increased by 274% and 314% compared to WT. The mutants showed 2.3 and 2.7-fold higher lipid productivity than the WT	Chang et al. (2020)
*Porphyridium purpureum*					
*P. purpureum CCMP 1328*	Chlorophyll synthase (*CHS*1)	Knockout	Cas9	*CHS*1 mutants produced significantly higher phycoerythrin compared to WT	Jeon et al. (2021)

### Chlamydomonas reinhardtii

**FIGURE 2:**
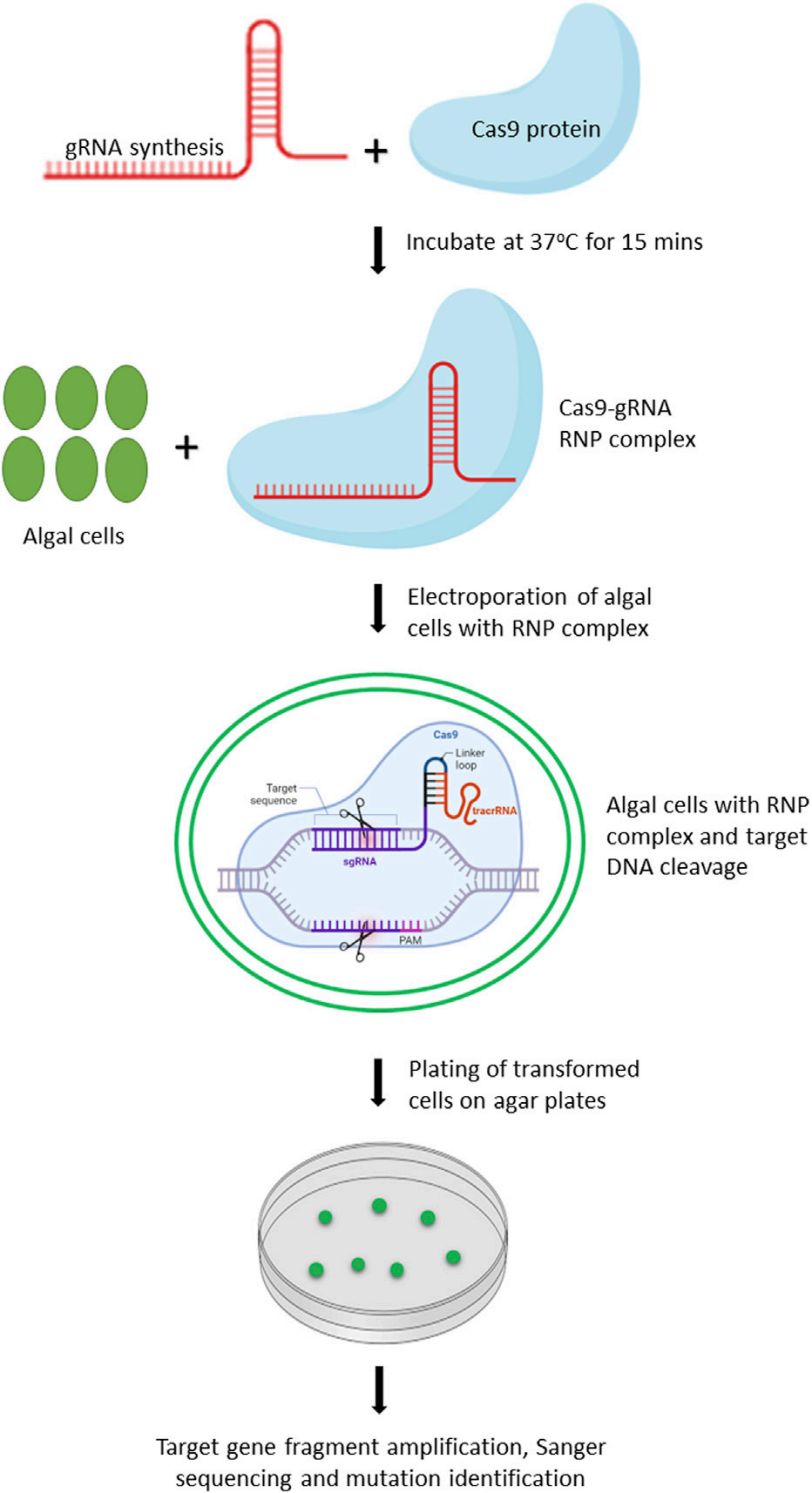
Schematic representation of workflow for editing target genes in microalgae by delivery of Cas9-gRNA RNP complex. Created with BioRender.com.

*Chlamydomonas reinhardtii* is an ideal model organism for bioengineering due to its well-characterized genome, ease of cultivation, and the applicability of extensive genetic tools for its modification. In 2014, Jiang et al. (2014) reported the first successful application of CRISPR/Cas9 in *C. reinhardtii*, demonstrating the feasibility of targeted gene editing in this organism. Since then, numerous studies have been conducted to further optimize and improve the CRISPR/Cas9 based editing in *C. reinhardtii* (Shin et al., 2016; Baek et al., 2018; Dhokane et al., 2020). Owing to the toxicity of constitutively expressed Cas9 protein and off-targeted mutation(s) associated with vector driven expression of Cas9, Shin et al. (2016) improved the editing efficiency by delivering gRNA-Cas9 ribonucleoproteins (RNPs) by 100-fold. We have provided the workflow for editing of target genes in microalgae using gRNA-Cas9 RNP complex (Figure 2).

In 2016, Shin et al. developed a high-throughput CRISPR/Cas9 system for *C. reinhardtii*, which enabled the editing of multiple gene targets simultaneously. This system was used to disrupt genes involved in the regulation of lipid metabolism, demonstrating the potential for using CRISPR/Cas9 for metabolic engineering in *C. reinhardtii* (Shin et al., 2016). The potential applications of gene knockout by CRISPR/Cas9 have been explored for modifying the biochemical pathways for accumulation of product(s) of interest.

Baek et al. (2016) showed the successful applicability of DNA-free RNA guided engineered nucleases RNPs by creating specific knockouts of *CpFTSY* gene with a 0.56% efficiency and zeaxanthin epoxidase (*ZEP*) gene with 0.46% efficiency. Baek et al. (2018) continued this study and knocked out *ZEP* gene in higher carotenoid production strain of *C. reinhardtii* CC-4349, resulting in 56-fold higher zeaxanthin levels without reduction in lutein levels. Feeding this mutant to hens also resulted in fortified eggs with higher lutein (2-fold) and zeaxanthin (2.2-fold) content (Baek et al., 2018).

In 2017, Kao and Ng tested the applicability of vector derived CRISPRi to downregulate the *CrPEPC1* gene, successfully manipulating the carbon flux to increase lipid production, and indicating that CRISPRi can be used for modulating the expression of target gene(s) in *C. reinhardtii* to improve desired traits (Kao and Ng, 2017).

In another study, Baek et al. reported the use of CRISPR/Cas9 system to edit the *C. reinhardtii* genome to increase the lipid content in cells. The authors targeted the genes encoding acyl-CoA diacylglycerol acyltransferase (*DGAT*) and phospholipid diacylglycerol acyltransferase (*PDAT*), which are involved in lipid biosynthesis and demonstrated that CRISPR/Cas9 system can be used to generate targeted mutations that increase the lipid content of *C. reinhardtii* (Baek et al., 2018).

Shin et al. (2019) used the RNP mediated CRISPR/Cas9 technology to increase the lipid content via knockout of the phospholipase A2 (*PLA2*) gene, increasing the diacylglycerol pool, and higher accumulation of triacylglycerol, without significantly effecting the growth of the cells.

An increase in lipid productivity was also observed by Nguyen et al. (2020) after the knockout of the *ELT* (Cre01.g000300) gene encoding an enzyme in lipid catabolism, resulting in 28.52% increased total lipid and a shift in the fatty acid composition with an increase of approximately 27.2% in the C18:1 proportion.

Song et al. (2020) used RNP mediated CRISPR/Cas9 technology to produce highly purified zeaxanthin by blocking the synthesis of lutein via knocking *ZEP* gene and α-branch of lycopene epsilon cyclase (*LCYE*) gene, leading to 60% higher zeaxanthin yield than parental strain. In a further study, Song et al. (2022) generated a double mutant by knocking out *ZEP* and the ADP-glucose pyrophosphorylase (*AGP*) genes to accumulate lutein (2.93 mg/g), zeaxanthin (3.12 mg/g), and lipids (450.09 mg/g) in an N-deprived condition, achieving an 81% higher oil productivity with increased macular pigment productivity.

Freudenberg et al. (2022) knocked out the key enzymes of polyamine biosynthesis pathway to study the accumulation of putrescine, demonstrating the crucial role of ornithine decarboxylase 1 (*ODC1*) as the limiting factor in putrescine accumulation. The group identified that overexpression of ornithine decarboxylases and functional knockout of amine oxidase 2 (*AMX*2) for preventing putrescine degradation resulted in a 10-fold increase in cellular putrescine titers and yielded 200 mg/L.

Overall, the use of CRISPR/Cas genome editing technology has greatly advanced the ability to manipulate the *Chlamydomonas* genome in a precise and efficient manner. These advancements have enabled researchers to study the functions of individual genes and engineer strains with desired traits for various applications.

### Phaeodactylum tricornutum

*Phaeodactylum tricornutum*, a marine diatom, has gained attention due to its unique characteristics, including rapid growth, potential to produce a variety of valuable bioactive compounds (e.g., pigments, lipids, and polysaccharides), and the concomitant assimilation of carbon dioxide. The lipid biosynthesis pathways of *P. tricornutum* have been extensively investigated and the species have been commercially exploited as a source of high-value carotenoids (fucoxanthin), omega-3 long-chain polyunsaturated fatty acids (LC-PUFA) and other lipids. These traits make it an attractive candidate for various biotechnological applications.

The CRISPR/Cas9 tool, for the first time, was successfully applied to *P. tricornutum* in 2016 (Nymark et al., 2016). The knockout mutants of chloroplast signal recognition particle 54 (*CPSRP54*) gene, a member of the chloroplast signal recognition particle pathway, were generated with a knockout efficiency of 31% (Nymark et al., 2016). After that, CRISPR-based gene knockout (Russo et al., 2018; Serif et al., 2018) and knockin (Moosburner et al., 2020) have been successfully reported in *P. tricornutum.*

In 2018, Russo et al. (2018) developed an episomal plasmid carrying Cas9 and transformed it via conjugation to strengthen CRISPR toolbox for *P. tricornutum*. The transformation of an episomal plasmid allowed to avoid unwanted perturbations due to random integration in the genome and excluding the Cas9 activity when it was no longer required, thereby reducing the probability of obtaining off-target mutations. Later in 2020, Moosburner et al. (2020) created knockout mutants by transforming episomal plasmid carrying Cas9 via particle-bombardment transformation method. The authors developed and reported a protocol that could select mutants in less than 3 weeks, demonstrating the feasibility of generating CRISPR-based industrial strains in minimal time. Furthermore, Taparia et al. (2022) successfully constructed a CRISPR/Cas9 episome for multiplexed targeting and creation of marker-free edited genomes. The group constructed an efficient, compact, episome expressing Cas9 targeting Stramenopile-type lipid droplet protein (*StLDP*) gene. The reported knockout efficiency ranged from 6.7% to 13.8%. This study provides a protocol for modular assembly of a multiplexed genome-editing episome that uses RNA polymerase II promoters, which can easily transcribe long sgRNA arrays and make multiplexed gene editing feasible.

Serif et al. (2018) first successfully demonstrated multiplexed genome-editing by transforming Cas9-gRNA RNPs using a gene gun in *P. tricornutum*. Two endogenous genes, *ptUMPS* (5-fluoroorotic acid resistance) and *PtAPT* (2-fluoroadenine resistance) were knocked out with 65%–100% efficiency, clearly showing the feasibility of multiplexed gene editing in this species (Serif et al., 2018). It opened the way to study the functions of multiple gene family members. Similarly, Stukenberg et al. (2018) further showed the efficiency of vector derived CRISPR/Cas9 with biolistic transformation by mutating the *vtc*2 and *Pho*4, observing the easiness of mono- and bi-allelic mutants and without any off targets in the genome. Later in 2021, Sharma et al. (2021) in *P. tricornutum* reported successful simultaneous knockout of five [light-harvesting complex (LHC)] homologous genes using two gRNAs and a high fidelity Cas9 nuclease in which four amino acids substitutions had been introduced compared to wild type (WT) Cas9 nuclease. This study clearly demonstrated that engineering Cas9 nuclease reduced off-target editing, indicating that the altered high fidelity Cas9 nuclease must be exploited for precise genome editing.

In a study, to investigate the function of cryptochrome *CryP* and its role in regulating fucoxanthin content, *CryP* gene was knocked out in *P. tricornutum* using CRISPR/Cas9. The authors reported that *CryP* knockout line demonstrated stable heredity after hundreds of generations. CryP functions as a blue light-sensitive protein that regulates the expression of genes encoding carotenoid biosynthesis enzymes and fucoxanthin chlorophyll *a*/c-binding proteins (FCPs). Upon knockout of the *CryP* gene, both fucoxanthin content and FCP levels in the *P. tricornutum* knockout line increased considerably compared to the WT (Yang et al., 2022).

Functional characterization of long-chain Acyl-CoA synthetases (LACS) isozymes by CRISPR/Cas9 knockouts of *ptACSL*1-5 genes was done by Hao et al. (2022). Their findings demonstrated the potential of generating gene knockout mutants with the mutation of large fragment deletion using multiplexed CRISPR/Cas9 and provided insights into the functions of LACS isozymes in lipid metabolism in the oleaginous microalgae (Hao et al., 2022).

CRISPR/Cas9 was also used to knockout genes in uracil, histidine, and tryptophan biosynthetic pathways using plasmid vector expressing Cas9 and target specific gRNA (Slattery et al., 2020). Sequencing of mutants indicated that editing events are characterized by the occurrence of large deletions of approximately 2.7 kb centered on the editing site. This study provides new auxotrophic markers to easily select mutants, a viable alternative to traditionally used antibiotic selection markers, thus aiding the development of marker-free production strains. Llavero-Pasquina et al. (2022) used CRISPR/Cas9 to test the predicted function of genes containing thiamine pyrophosphate riboswitches. Knockout mutants of violaxanthin de-epoxidase (*VDL*2) and zeaxanthin epoxidase (*ZEP*1) were developed in *P. tricornutum* via CRISPR/Cas9, demonstrating their role in fucoxanthin biosynthesis pathways via xanthophyll cycle (Bai et al., 2022). This study clearly elucidates that CRISPR can be efficiently used to knockout one or multiple genes simultaneously and demonstrate their functions in different biosynthetic pathways.

Recently, *P. tricornutum* fucosyltransferase 1 (*PtFucT*1) which is located on the medial/trans-Golgi apparatus was knocked out using CRISPR/Cas9 (Xie et al., 2023). The knockout mutants of *PtFucT*1 demonstrated reduced algal growth, biomass, and photosynthetic efficiency. Golgi fucosyltransferase 1 (*PtFucT*1) revealed its important role in α-1,4-fucose modification of N-glycan. This study provided critical information to understand the mechanism of protein N-glycosylation modification and demonstrated *P. tricornutum* as an alternative, ecofriendly cell factory to produce biopharmaceuticals (Xie et al., 2023).

Understanding the regulatory mechanism of neutral lipid accumulation and degradation, which is mediated by lipid droplet-associated proteins, is important in improving lipid productivity. Hence, a knockout mutant of Stramenopile-type lipid droplet protein (*StLDP*) was generated by CRISPR/Cas9 based genome editing (Yoneda et al., 2023). The mutant strain showed a decrease in lipid droplet (LD) numbers per cell, an increase in LD size, and no alteration of neutral lipid content under nitrogen deficiency, clearly elucidating that *StLDP* acts as an LD scaffold protein.

### *Nannochloropsis* spp.

*Nannochloropsis* spp. are the emerging industrial microalgae favored due to their robust growth performance at large scale, accumulation proteins, and triacylglycerol with high-value PUFAs (Wang et al., 2021). They serve as an outstanding research model for synthetic biology owing to their small genome size, simple gene structure, and accessibility to newly developed genetic tools. The resources for genomic, transcriptomic, proteomic, lipidomic, and physiological data are extensive in this species, and thus, can be exploited for targeted genome engineering (Wang et al., 2021).

First study on application of CRISPR/Cas9 in *Nannochloropsis* spp. was reported in 2016, by creating knockout mutants of *nitrate reductase* gene via plasmid-based expression of Cas9 (Wang et al., 2016). This demonstration of CRISPR/Cas9-based genome editing in *Nannochloropsis* opened the doors for *Nannochloropsis*-based biotechnological applications. Later, in 2017, the expression of a transcription regulator *ZnCys* in *Nannochloropsis gaditana* CCMP1894 was attenuated by insertion in the 5′UTR region using Cas9, which enabled to double the strain’s lipid productivity while retaining its ability to grow and fix CO_2_ at levels nearly equivalent to those of the WT strain, under dense semicontinuous culture (Ajjawi et al., 2017). This ability of CRISPR/Cas system to control the production of molecules of interest will more likely enable the commercialization of microalgal-derived bioproducts.

To broaden the CRISPR toolbox for *Nannochloropsis* spp., an episomal plasmid based Cas9 system was developed and transformed in *N. oceanica* CCMP1779 (Poliner et al., 2018). This system efficiently generated targeted mutations and allowed the loss of episomal DNA after the removal of selection pressure, resulting in marker-free nontransgenic engineered lines (Poliner et al., 2018). The generation of such non-transgenic mutant strains may face less regulatory challenges and more likely to meet acceptability in various markets.

Later in 2018, Verruto et al. (2018) reported unrestrained markerless trait stacking in *Nannochloropsis gaditana* through combined genome editing and marker recycling technologies. The authors demonstrated the proof-of-concept for generation of a markerless knockout in a gene encoding an acyl-CoA oxidase (*Aco*1) as well as the markerless recapitulation of a 2-kb insert in the *ZnCys* gene 5′-UTR that resulted in a doubling of lipid productivity in WT strain (Verruto et al., 2018). In the same study, they generated mutants that exhibit approximately 50% reduction in photosynthetic antennae size by markerless knockout of seven genes in the large light-harvesting complex gene family (Verruto et al., 2018).

In 2020, Jeong et al. (2020) used CRISPR/Cas9 to knockout three genes (*cesA*1, 2, and 4) involved in cellulose biosynthesis. Cell wall thickness and cellulose content were reduced in the *cesA*1 mutant, but not in *cesA*2 or *cesA*4. The *CesA*1 mutation resulted in a reduction of chrysolaminarin and neutral lipid contents by 66.3% and 37.1%, respectively, but increased the soluble protein content by 1.8-fold. Further, cells with a thinned cell wall were susceptible to mechanical stress, resulting in a 1.7-fold enhancement of lipid extractability. This study clearly demonstrated CRISPR tool could efficiently led to the production of chassis strain for different industrial applications.

In another study, Naduthodi et al. (2021) used Cas12a nuclease (variant of Cas9) in *Nannochloropsis oceanica* for precise modifications. They demonstrated RNPs and homology-directed repair (HDR) genome editing strategy to generate scarless and markerless mutants. The authors also developed an episomal plasmid based Cas12a system for efficiently introducing indels at the target site. They also reported the ease of multiplexed genome engineering using Cas12a. Furthermore, dCas9 and dfnCas12a based CRISPRi platform was reported for down regulating of target genes. Reduction of 85% in the transcript levels upon performing CRISPRi with dCas9 in *N. oceanica* was reported. Overall, these developments substantially accelerate genome engineering efforts in *N. oceanica* and potentially provide a general toolbox for improving other *Nannochloropsis* strains for industrial applications (Naduthodi et al., 2021).

A study was conducted to build a minimal genome for *Nannochloropsis using* CRISPR/Cas9 system by serially and precisely deleting large genome fragments of approximately 100 kb from its 30.01 Mb nuclear genome. The “non-essential” chromosomal regions based on minimal gene expression [low expression regions (LERs)] under N-replete and N-depleted conditions were identified and deleted. The LER1 deletion (∼110 kb deletion) and the LER1–LER2 serial deletion (∼214 kb in total) showed essentially normal growth, lipid contents, fatty acid saturation levels, and photosynthesis, or, in the case of a LER1–LER2 double-deletion mutant, slightly higher growth and biomass productivity than the WT (Wang et al., 2021).

**FIGURE 3:**
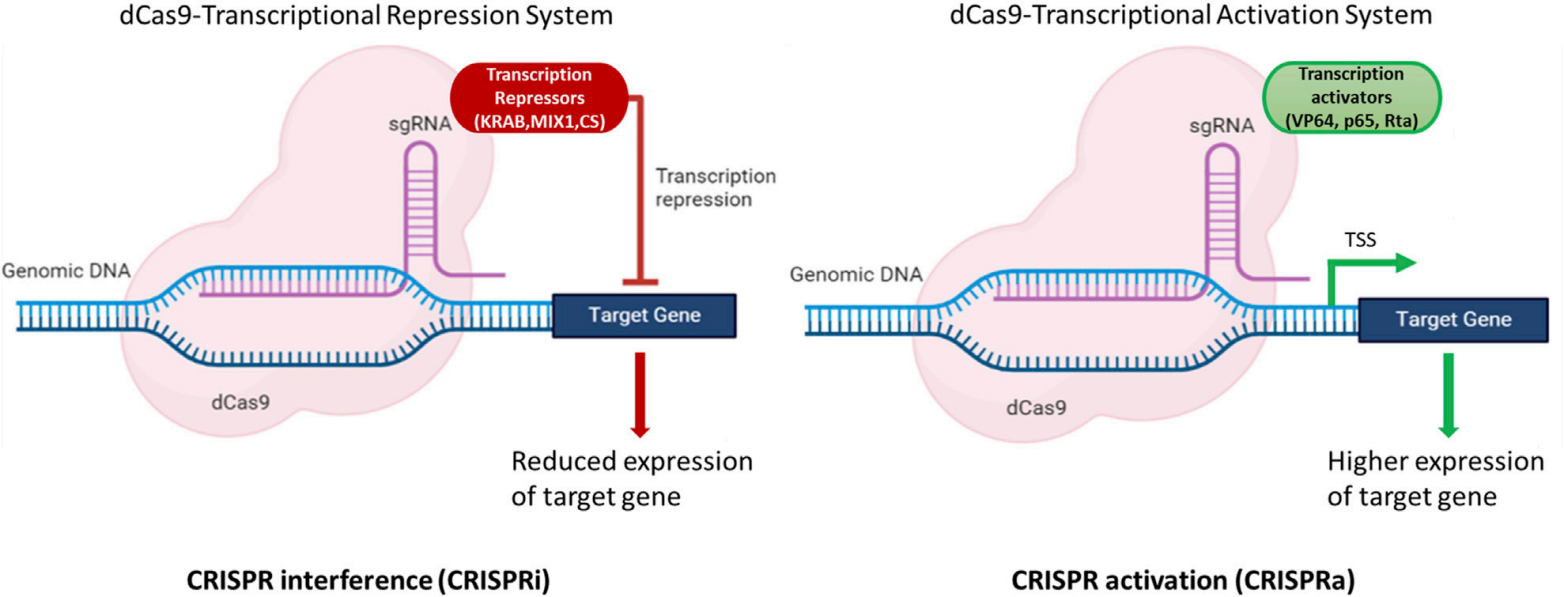
Illustration of dCas9 based expression modulation of target genes. The dCas9-gRNA complex fused with effectors, either transcriptional activators (VP64, p65, Rta) or repressors (KRAB, MIX1, CS) bind to the upstream regulatory elements and modulate the expression of target gene(s). CRISPR interference (CRISPRi) is used to repress the gene(s) of the competing pathways to channelize the flux towards the molecule(s) of interest. CRISPR activation (CRISPRa) is used to enhance the expression of the target gene(s) and hence aid in improving the productivities of target molecules of interest. Created with BioRender.com.

A transcriptional activation system based on CRISPR/dCas9 in *N. oceanica* IMET1 was constructed in a study by Wei et al. (2022). This construct (dCas9 protein fused with transcriptional activator VP64) could efficiently alter gene expression. The expression of *g1248* gene responsible for DNA or RNA methylation as a methyltransferase was increased by 2–6 fold at the transcriptional level. Furthermore, the growth and photosynthetic parameter (Fv/Fm) of mutants was increased by 23% and 12%, respectively, compared to WT under atmospheric CO_2_ concentration (Wei et al., 2022). This study clearly demonstrated that the expression of target genes could be modulated using dCas9-based CRISPR system in *Nannochloropsis* spp. to achieve higher titers of molecules of interest. These strategies can aid in the development of a strain that can produce higher titers of biomolecules of interest either by modulating gene expression by CRISPRa or CRISPRi or by knocking out the genes of the competing pathways (Figure 3).

### *Chlorella* spp.

*Chlorella* species have easier culture conditions, rapid growth rate, ability to thrive in varied and challenging environments, and produce diverse biomolecules. *Chlorella* spp. are rich in lipids, making it an excellent candidate for biofuel production. They are rich in nutritional compounds that include high protein, essential fatty acids, vitamins, and minerals content. They are widely used as a dietary supplement and functional food ingredient due to their potential health benefits, such as immune system support, detoxification, and antioxidant properties. However, insufficient genomic resources for *Chlorella* and low transformation efficiency are the major concerns for genetic improvement of these genus.

The first report on successful application of CRISPR-based gene editing in *Chlorell*a spp. was reported in 2020 (Lin and Ng, 2020). In this study, a plasmid vector harboring Cas9 and omega-3 fatty acid desaturase (*fad*3) gene specific gRNA was transformed in *Chlorella vulgaris* FSP-E, the knockout mutants of *fad*3 showed 46% (w/w) higher lipid content over WT (Lin and Ng, 2020). The study clearly demonstrated relevant empirical evidence of applying CRISPR-based genetic manipulation in *Chlorella* spp. for producing robust chassis strains of *Chlorella* for several industrial applications.

Knockout mutants of two genes, nitrate reductase (*NR*) and adenine phosphoribosyltransferase (*APT*), were generated in *C. vulgaris* UTEX395 using plasmid harboring Cas9 and target specific gRNAs and ribonucleoprotein complex (Kim et al., 2021). The mutants were negatively selected on potassium chlorate or 2-fluoroadenine clearly exhibiting the practicality of generating markerless mutants in *Chlorella* that might likely not fall under regulatory purview (Kim et al., 2021).

Lin et al. (2022) used CRISPRa/i system to modulate the gene expression in *Chlorella sorokiniana* UTEX 1602. They demonstrated that gene regulation via dCas9-VP64 (CRISPRa) increased the protein contents by approximately 60% (w/w), while that via dCas9-KRAB (CRISPRi) increased the protein content to 65%, and lipid accumulated in the range of 150–250 mg/L (WT: 150 mg/L). This study clearly opens new avenues in altering the expression levels of target genes, thus allowing improvement in the titer yields of target molecules.

### *Picochlorum* spp.

*Picochlorum* genus have been explored to produce various biomolecules, including lipids, pigments, proteins, and bioactive compounds. For example, lipids from *Picochlorum* spp. can be utilized to produce biofuels or high-value fatty acids. Pigments derived from these algae, such as chlorophylls and carotenoids, have applications in the food, cosmetic, and pharmaceutical industries. Additionally, *Picochlorum* spp. have the potential to produce recombinant proteins and enzymes*.* Green algae in *Picochlorum* genus can achieve rapid growth rates and thrive in intense light, high-temperature, and high-salt conditions.

In 2020, for the first, time CRISPR/Cas9 was applied in *Picochlorum celeri* to generate knockout mutants of two genes, nitrate reductase and carotenoid isomerase. The loss-of-function mutants were present in approximately 6% of transformants targeting nitrate reductase and in approximately 50% of transformants targeting carotenoid isomerase (Krishnan et al., 2020). This report provides evidence that CRISPR-based genome editing is feasible in *Picochlorum* spp. that can rapidly generate mutants, help in elucidating the functions of candidate genes, and precisely manipulate genes/metabolic pathways to produce robust production strains.

## Key considerations for using CRISPR-based genome editing in microalgae

### Transformation method

One of the major challenges is the lack of efficient delivery methods for genome editing components into microalgae cells. As with other eukaryotic cells, delivery of foreign nucleic acids into microalgae cells is challenging due to the presence of cell wall and low efficiency of transformation methods. Various delivery methods, such as electroporation, biolistic transformation, and *Agrobacterium*-mediated transformation, have been developed for microalgae (Radakovits et al., 2010), but their efficiency varies widely among different species and strains. Thus, well optimized and reproducible transformation methods need to be implemented to achieve high levels of targeted modifications.

### Homologous recombination

Low efficiencies of homologous recombination impede precise targeted knockin of desired gene fragments in microalgae. Even after several optimizations, such as concentrations of DNA fragments, lengths of HDR cassette(s), and optimizing the transformation conditions, the success of homologous recombination has been very low in *C. reinhardtii* (Plecenikova et al., 2013) and *Phaeodactylum* (Moosburner et al., 2020). The codelivery of gRNA-Cas9 RNP complex along with a dsDNA repair template efficiently enhanced homologous recombination at the target site, resulting in a remarkable higher percentage of targeted integration clones (Naduthodi et al., 2019). Very recently, with optimizing the concentrations of gRNA and Cas9 protein and with 1000-bp homology arms, the targeted knockin efficiency was reported to be 15% in *C. reinhardtii* (Shahabadi et al., 2023). However, the efficiency of HDR is species-dependent, and it has been proposed that the homologous recombination efficiency in microalgae could more likely be enhanced by interfering with the DNA repair proteins Ku70, Ku80, DNA ligase IV, and other relevant genes (He et al., 2013). Hence, considering the native efficiency of homologous recombination in species of interest, several parameters need to be considered, such as concentration of HDR cassette, length of homology arms, site of integration, concentrations of gRNA and Cas9 nuclease, and the best optimized transformation method, before planning targeted knockin experiments.

### Off-targets

Another major concern is the off-target mutations caused by genome editing tools. Although CRISPR/Cas has been shown to have high specificity, it can still induce unintended mutations at off-target sites (Hsu et al., 2013). This can be particularly problematic in microalgae, where the genomic organization and presence of repetitive sequences can increase the likelihood of off-target effects. The targeting specificity depends on how guide RNA(s) are designed to target the gene of interest, the type of nuclease, and the protospacer adjacent motif (PAM) recognition sequences. Different tools such as Cas-OFFinder, Benchling, CHOPCHOP, and CRISPOR are developed to design target specific gRNAs and predict off-target cleavage sites (Ghribi et al., 2020). Unavailability of high-quality whole genome sequence makes identification/prediction of the off-target sites challenging. Therefore, it is very important that gRNAs are designed carefully to minimize or have no-off targets, wherever possible.

### Use of high-fidelity nucleases

In recent years, extensive studies have been conducted to improve the gene-editing specificity of the most popular Cas nucleases using different strategies. Non-rational strategies like directed evolution and rational strategies such as structure and/or function-guided protein engineering or combination of both are used for engineering high-fidelity Cas proteins and representative variants (Huang et al., 2022). HiFi Cas9, xCas9, SpartaCas, efSaCas9 (enhance-fidelity SaCas9), SpCas9 nickase, SpCas9-D1135E, eSpCas9, eSaCas9, and SaCas9-HF are some of the high-fidelity variants that have been discovered through these strategies (Huang et al., 2022). Engineered Cas9 variants, Cas9 homologs such as KKH SaCas9 (smaller than SpCas9) (Ran et al., 2013), and novel Cas9 protein such as C2C1 (Shmakov et al., 2015) have been successfully applied in genome editing with high efficiency and specificity. It was reported in a study that the DNA binding specificity of a catalytically inactive Cas9 mutant (dCas9) was sufficiently high in *E. coli*, yielding no detectable off-target transcriptional repression in the *E. coli* transcriptome (Qi et al., 2013). Apart from SpCas9 variants, other Cas proteins such as *Staphylococcus aureus* Cas9 (SaCas9), with different PAM requirements is now widely used for gene editing applications due to its high activity in eukaryotic systems (Wang et al., 2019). Recent advancements in the discovery and/or development of several HiFi variants of nuclease(s) suggest that the specificity of WT-nucleases can be enhanced to a greater extent more likely using protein engineering or evolution approaches. Use of such HiFi nucleases will greatly facilitate enhanced editing efficiencies.

### Sequencing of CRISPR edited clones

It is well reported that editing might also occur at unintended loci if there is sequence complementarity with the designed gRNAs in the genome. Identifying these off-target sites using computational analysis and analyzing the off-target editing becomes very crucial to verify, if these edits lead to any unintended effects. Whole genome sequencing of the edited clone using next-generation sequencing (NGS) platforms is apparently the only way to discover off target editing. NGS technologies also aid in identification of allelic changes in CRISPR edited clones (Veeranagouda et al., 2018). A NGS method called CRISPR/Cas9 edited site sequencing (CRES-Seq) has been reported for efficient and high throughput screening of CRISPR/Cas9 edited clones. CRES-Seq aids in precise genotyping up to 96 CRISPR-Cas9-edited sites in a single MiniSeq (Illumina) run with an approximate sequencing cost of $6/clone (Veeranagouda et al., 2018). Alternatively, due to unavailability of funds, checking for off-target editing is done by performing sequencing of sequences with the highest probability of off-target effects (i.e., most like your CRISPR target region). The targeted CRISPR induced mutations can be confirmed by amplifying the target regions and sequencing the purified fragments using Sanger sequencing (Clement et al., 2020). While sequencing the fragment using Sanger method make sure, you sequence each purified fragment atleast twice, to ensure you do not have erroneous sequenced data. Moreover, always send purified PCR fragments of targeted regions for Sanger sequencing, as the purity of samples will determine the quality of sequenced data. It is always advisable to have an amplicon size of 600–700 bp for Sanger sequencing and oligos that are used for sequencing must bind at least 110 bp away from the cleavage sites, in case of multiple gRNAs used for targeting the same region.

### Regulatory challenges

In addition to the technical limitations described in previous sections, the regulatory framework for the use of genome editing tools in microalgae is still evolving. In many countries, the use of genome editing tools in agriculture and biotechnology is subject to strict regulations and the safety and ethical implications of their use are still being debated. The rapid emergence and ongoing developments in genome editing demand a timely review and revision of the current definitions and regulations around genetically modified organisms (GMOs) (Spicer and Molnar, 2018). New strains of microalgae generated through gene editing, where the genetic manipulations are made only in the native genes are also considered under GMO umbrella in the European Union, hampering practical and commercial applications. In March 2022, the Ministry of Environment, Forest and Climate Change, Government of India issued an office memorandum for exemption of genome-edited plants that falls in SDN1 (site directed nuclease 1) and SDN2 categories, from the Indian GMO regulation (https://pib.gov.in/PressReleasePage.aspx?PRID=1871153#:∼:text=Ministry%20of%20Environment%2C%20Forest%20and,Rules%201989%20of%20EPA%2C%201986). SDN1 and SDN2 includes alterations only within the organisms own genetic code. In the US, the US Department of Agriculture are imposing regulations on the product or the organism itself rather than the process, which tends to favor gene-edited organisms for their commercial applications (Spicer and Molnar, 2018). Therefore, the regulatory landscape for gene edited organisms needs to be clearly understood before initiating projects involving application of genome editing tools.

## Conclusion

Microalgae are attractive candidates for bioengineering due to their high photosynthetic efficiency and higher growth rates. They are increasingly favored sustainable feedstock for food, feed, biofuels, and nutraceutical applications. Despite the apparent advantages, the major bottleneck in employing microalgae as an economically viable production platform is the high cost of biomass production. Genetic engineering has an ability to change the inherent potential of the strain for improved productivity by augmenting various genetic traits. CRISPR/Cas9 allows precise and targeted genetic manipulations literally at any loci in the genome. CRISPR-aided bioengineering has emerged as a powerful tool and is being widely applied for the genetic manipulation of microalgae for enhanced performance. CRISPR/Cas systems have been successfully used in several microalgal species, including *Chlamydomonas reinhardtii*, *Nannochloropsis* spp., *Chlorella* spp., *Phaeodactylum tricornutum* and many more. Overall, CRISPR-aided bioengineering has enormous potential for the development of microalgae-based bioproduction platforms to produce a wide range of value-added products. However, further research is needed to optimize the use of CRISPR/Cas tools in microalgae to overcome the associated challenges.
